# Omentin-1 in diabetes mellitus: A systematic review and meta-analysis

**DOI:** 10.1371/journal.pone.0226292

**Published:** 2019-12-10

**Authors:** Xiongfeng Pan, Atipatsa C. Kaminga, Shi Wu Wen, Kwabena Acheampong, Aizhong Liu

**Affiliations:** 1 Department of Epidemiology and Health Statistics, Xiangya School of Public Health, Central South University, Changsha, China; 2 Department of Mathematics and Statistics, Mzuzu University, Mzuzu, Malawi; 3 Department of Obstetrics and Gynaecology and Ottawa Hospital Research Institute, University of Ottawa, Ottawa, Ontario, Canada; 4 Department of Public, School of Postgraduate Studies, Adventist University of Africa, Nairobi, Kenya; Karolinska Institutet, SWEDEN

## Abstract

**Objective:**

Previous studies found inconsistent results on the relationship between diabetes mellitus and concentrations of omentin-1. This study performed a systematic review and meta-analysis to summarize previous findings on this relationship.

**Methods:**

Studies related to this outcome were obtained using a systematic search in the electronic databases of Cochrane Library, PubMed, Embase, SCOPUS, Google Scholar, gray literature and Web of Science in September 2019. The random effects model was used to measure the strength of the association between diabetes mellitus and concentrations of omentin-1, using standardized mean difference.

**Results:**

Forty-two eligible studies were included in the final meta-analysis. There was no significant difference in omentin-1 concentration between patients with type 1 diabetes mellitus and the controls. On the other hand, lower concentration levels of omentin-1 were observed in patients with gestational diabetes mellitus (standardized mean difference:-0.44, 95% confidence interval:-0.76; -0.12, p = 0.007), or type 2 diabetes mellitus (standardized mean difference: -1.74, 95% confidence interval: -2.31; -1.16, p< 0.001) than in the controls.

**Conclusion:**

Decreased omentin-1 concentrations may be an important indicator for gestational diabetes mellitus and type 2 diabetes mellitus. More studies are needed to validate this hypothesis and evaluate the role of omentin-1 concentrations in type 1 diabetes mellitus.

## Introduction

Diabetes mellitus (DM) is characterized by a set of metabolic disorders which are related to high blood sugar levels over a lengthy interval of time and may cause many complications [1]. DM is classified into three types: gestational diabetes mellitus (GDM), type 1 diabetes mellitus (T1DM), and type 2 diabetes mellitus (T2DM) [2, 3]. The development of DM has been a prominent global public health issue [4]. However, it has been observed that the etiology of DM is complex, in the sense that it is influenced by numerous genetic, life-styles, psychosocial, and other environmental factors [5].

Recently, many adipocyte-secreted proteins as well as adipokines have been introduced as novel links to DM [6]. It is widely accepted that the adipokines participate in many metabolic processes, including energy expenditure, appetite control, insulin sensitivity, and regulation of adipogenesis [7, 8].

Omentin-1, an important adipokine, is secreted from the visceral fat adipose tissue [9]. Evidence has shown that, compared to subcutaneous obesity, Omentin-1 is more influential in the prognosis of DM. As regards its development, Omentin-1 is made by vascular cells of visceral fat adipose tissue, when it exerts its actions in the manner as endocrine, paracrine, and autocrine [10]. It is well known that there are direct and indirect hypothetical mechanisms that play an important role in it. Moreover, these hypothetical mechanisms suggest that altered omentin-1 secretion might changes into glucose homoeostasis, and subsequently contribute to the development of diabetes [11].

Overall, a substantial number of recent studies on the association between concentrations of omentin-1 and DM found contradictory results. For example, concentrations of omentin-1 were lowered in patients with DM in many clinical studies [12]. However, other studies find that DM had higher concentration levels of omentin-1 than controls [13]. Although a previous meta-analysis analyzed the relationship between concentrations ometin-1 and diabetes mellitus, that study focused only on T1DM and T2DM, and included only 28 articles. Therefore, it was necessary to conduct a more comprehensive systematic review and meta-analysis of this relationship [14]. Accordingly, the aim of this study was to use systematic review and meta-analysis methods to synthesize research results related to the association between omentin-1 concentrations and DM, and to measure the significance of this association.

## Methods

Ethics Statement: All analyses were based on previous published studies; thus, no ethical approval and patient consent are required.

This systematic review and meta-analysis was performed following the guidelines as outlined in the Cochrane handbook version 5.1.0, and results were presented with respect to the Preferred Reporting Items for Systematic Reviews and Meta-Analyses (PRISMA) checklist [15]. Meanwhile, the study is registered with International Prospective Register of Systematic Reviews PROSPERO (CRD42019126305).

### Criteria for selecting studies

Studies were included in this meta-analysis only if they met the following selection criteria: (1) case-control or cross sectional studies; (2) studies reported the DM diagnostic criteria; (3) studies reported mean and standard deviation (SD) of the concentrations of omentin-1 (alternatively author or authors of studies could provide these values upon request); (4) studies were peer-reviewed publications; and (5) studies were published in English before September 2019 in the specified electronic databases. The exclusion criteria were (1) studies were review articles or case reports; (2) studies studied DM in the occurrence of other metabolic diseases; (3) studies indicated that omentin-1 was pharmacologically challenged before it was measured; and (4) studies did not study humans.

Two authors [KA and AK] independently assessed the study articles for eligibility. If there were any discrepancies between them, the final decision was arrived at by discussion with the third author [SW].

### Literature search

Keywords related to the topic of this study were used to search relevant research articles in the following electronic bibliographic databases: Cochrane Library, PubMed, Embase, SCOPUS, Google Scholar, gray literature and Web of Science. All different possible combinations of the keywords for diabetes mellitus and omentin-1 in the title or abstract field were considered in the search strategy. Detailed search strategies are described in the S1 Table. For example, in Embase database, the search was run as follows: ('diabetes mellitus':ab,ti OR 'diabetes':ab,ti OR 'mellitus':ab,ti OR 'diabetic':ab,ti OR 'diabete':ab,ti OR 'glycuresis':ab,ti OR 'diabetics':ab,ti OR 'type 2 diabetes':ab,ti OR 't2d':ab,ti OR 'type 2 diabetes mellitus':ab,ti OR 't2dm':ab,ti OR 'insulin-dependent diabetes mellitus':ab,ti OR 'iddm':ab,ti OR 'juvenile diabetes':ab,ti OR 'non insulin-dependent diabetes mellitus':ab,ti OR 'niddm':ab,ti OR 'adult-onset diabetes':ab,ti OR 'type 1 diabetes':ab,ti OR 't1d':ab,ti OR 'type 1 diabetes mellitus':ab,ti OR 't1dm':ab,ti OR 'gestational diabetes':ab,ti OR 'gestational diabetes mellitus':ab,ti OR 'gdm':ab,ti) AND ('omentin':ab,ti OR 'itln1 protein':ab,ti OR 'intelectin':ab,ti OR 'omentin-1':ab,ti OR 'intelectin-1':ab,ti).

**Fig 1:**
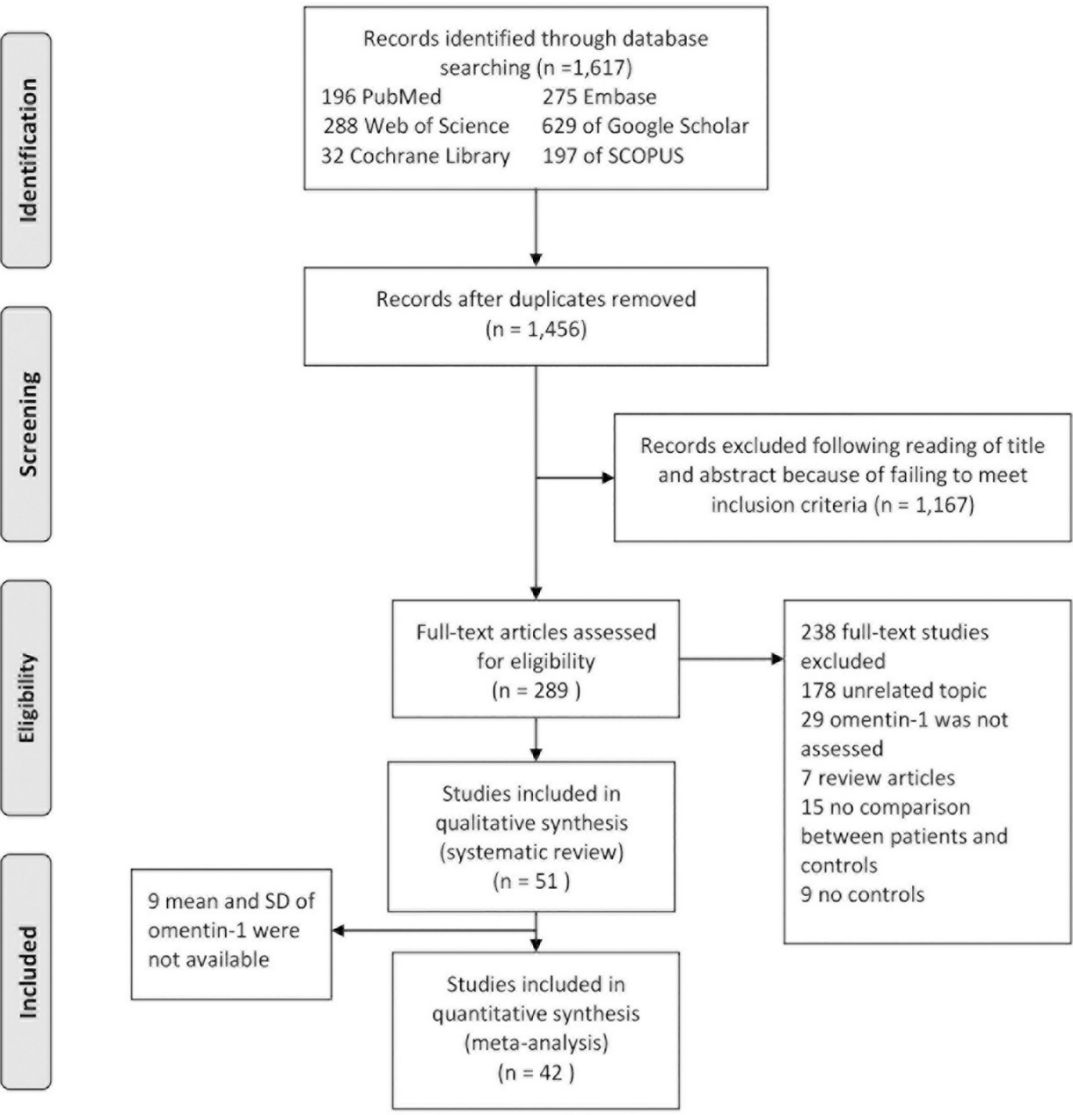
Flowchart of study selection. Showing the process by which relevant studies were retrieved from the databases, assessed, and selected, or excluded. Preferred reporting items for systematic reviews and meta-analyses (PRISMA) diagram for study search.

Initially, titles and abstracts of the study articles retrieved from the databases were assessed against the study inclusion criteria. Thus, two authors [KA and AK] independently made this assessment to identify potentially relevant study articles for the full-text review. The third author [AL] helped to resolve any discrepancies between KA and AK on their choices of potentially relevant studies. Again, two reviewers [XP and KA] independently conducted the full-text review as appropriate and identified the final set of eligible articles for this systematic review and meta-analysis. Fig 1 shows the search and screening process.

### Extraction of relevant data

Data were extracted according to a predesigned data extraction form depicting data related to the following variables: (1) first author’s last name and publication year; (2) study design and study country; (3) characteristics of the sample such as DM type, and mean concentration of omentin-1 and its standard deviation (mean, SD); (4) characteristics of the sample such as Body Mass Index (BMI), mean age and its SD, and gender; and (5) collection of omentin-1 samples and assay methods, and storage temperatures. EpiData 3.0 was used to organize these specified data.

### Study quality assessment

Quality of studies was assessed by the Newcastle-Ottawa Scale (NOS) [16]. It is a nine-star rating system designed for non-randomized studies, which contains three domains and eight items. The three domains consist of the following broad perspectives: (1) Selection; (2) Comparability; and (3) Outcome. Based on the range of scores, 7–9, 4–6, and 0–3, studies are graded as high, moderate, and low quality, respectively. This assessment was done in this study to determine the strength of scientific evidence.

### Statistical analysis

First, meta-analyses of the association between omentin-1 concentrations and DM were performed, while taking all the specified variables into account, by comparing these concentrations between patients with DM and the controls. Basically, the standardized mean difference (SMD) was used to assess the effect size of the concentrations on DM, simply because the included studies varied in their methodologies when measuring concentrations of omentin-1. In this regard, the SMD was calculated as Cohen’s d [17]. Heterogeneity among the eligible studies was assessed using the I^2^ statistic, which was validated by the H value and its 95%CI [18]. The H value of 1 indicates no heterogeneity, >1.5 indicates heterogeneity, <1.2 indicates homogeneity, and when H is between 1.2 and 1.5 such that the corresponding 95% CI does not contain 1, then there is heterogeneity. Considering the I^2^ statistic, the values of 75%, 50%, and 25%, represent statistically high, moderate, and low degrees of heterogeneity [19]. Furthermore, subgroup analyses were performed to see if some characteristics related to the eligible studies could explain sources of heterogeneity. The model used to synthesize the related data for this study was the random effects model, which suited well considering the varying populations and criteria for defining outcomes across the eligible studies [20]. Also, in order to see if the stability of the results of this study could be affected by a study or cluster of studies with a common set of characteristics, sensitivity analysis was conducted. In this analysis the meta-analysis was repeated every time each study was omitted in turn. Publication bias, which may exist when selecting studies for systematic reviews, was assessed using symmetry of funnel plots for at least 10 studies which reported primary outcomes. The significance of the publication bias was ascertained using Egger’s linear regression test [21]. All meta-analyses were performed by the ‘meta’ and ‘metafor’ package in R software (version R 3.4.2). The statistical significance in all the statistical tests was set at the 5% level, and all the statistical tests were two-sided.

## Results

### Literature search

Fig 1 illustrates flow chart of the study selection process. A total of 1,617 articles were identified from the six databases as follows: 196 from PubMed, 275 from Embase, 288 from Web of Science, 629 from Google Scholar, 32 from Cochrane Library and 197 from SCOPUS. Of the 1,617 articles, the abstracts of the 1,456 articles went through further assessment, which resulted in exclusion of 1,167 studies for not meeting the inclusion criteria. The full text of the remaining 289 articles were reviewed and this resulted in the exclusion of 178 articles for having irrelevant topics, 29 for not assessing omentin-1, 7 for being review articles, 15 for not comparing patients and controls, and 9 for not reporting results for controls. At last, 51 articles satisfied the inclusion criteria for systematic review and 42 articles satisfied the inclusion criteria for meta-analysis, hence were used for this study.

### Characteristics of eligible studies

Table 1 presents the general characteristics of the 51 eligible studies. All these eligible studies were published between 2008 and 2019. Forty-two studies analyzed omentin-1 concentrations using serum samples and nine studies analyzed omentin-1 concentrations using plasma samples. Nine of the studies were conducted in Egypt, six in Poland, six in China, five in Turkey, and three in Germany. There were two studies from each of the following countries: India, Iran, Italy, Jordan and Czech Republic. Also, there was one study from each of the following countries: Australia, Austria, Canada, Croatia, Denmark, Greece, Japan, Korea, Netherlands, Spain and Thailand, respective. For different types of DM, 6 studies studied GDM, 3 studies studied Prediabetics, 6 studies studied T1DM, and 36 studies studied T2DM. Conflicting results were notable among the eligible studies. For example, 6 studies showed that people with diabetes had significantly higher omentin-1 concentrations than the controls, 30 studies showed that people with diabetes had significantly lower omentin-1 concentrations than the controls, and 6 studies showed that there was no significant difference in omentin-1 concentrations between diabetes patients and the controls. In addition, other studies showed that intervention measures such as diet plus metformin could reduce omentum protein levels in patients with T2DM. Table 2 presents the attributes of the 42 eligible studies for meta-analysis. Altogether, these studies compared omentin-1 concentrations between 2,199 T2DM patients and 2,043 controls; 406 T1DM patients and 357 controls; and 256 GDM patients and 312 controls. The NOS scores of these studies varied between 5 and 8, with 26 studies graded as high quality and 16 as moderate quality. Detailed study quality assessments are described in S4 Table.

**Table 1:** Characteristics of the studies included for the omentin-1 concentrations in diabetes mellitus.

Study	Material	Country	Type	Outcome
Abd-elbaky 2015	Serum	Egypt	T2DM	Lower level of omentin-1 was observed in patients with diabetes mellitus than in the controls
Abdelraoufkorany 2018	Serum	Egypt	T2DM	Lower level of omentin-1 was observed in patients with diabetes mellitus than in the controls
Abell 2017	Serum	Australia	GDM	Lower level of omentin-1 was observed in patients with diabetes mellitus than in the controls
Ahmed 2018	Serum	Egypt	T2DM	Lower level of omentin-1 was observed in patients with diabetes mellitus than in the controls
Akbarzadeh 2012	Plasma	Iran	T2DM	Lower level of omentin-1 was observed in patients with diabetes mellitus than in the controls
Akour 2016	Serum	Jordan	T2DM	Lower level of omentin-1 was observed in patients with diabetes mellitus than in the controls
Cai 2008	Serum	China	T2DM	Lower level of omentin-1 was observed in patients with diabetes mellitus than in the controls
Dayem 2015	Serum	Egypt	T1DM	Lower level of omentin-1 was observed in patients with diabetes mellitus than in the controls
El-mesallamy 2011	Serum	Egypt	T2DM	There was no significant difference in Omentin1 level between diabetic patients and control group.
Elsaid 2018	Serum	Egypt	T2DM	Lower level of omentin-1 was observed in patients with diabetes mellitus than in the controls
Franz 2018	Plasma	Austria	GDM	There was no significant difference in Omentin1 level between diabetic patients and control group.
Greulich 2013	Plasma	Netherlands	T2DM	Lower level of omentin-1 was observed in patients with diabetes mellitus than in the controls
Hayashi 2018	Serum	Japan	T2DM	Higher level of omentin-1 was observed in patients with diabetes mellitus than in the controls
Herder 2017	Serum	Germany	T2DM	Higher level of omentin-1 was observed in patients with diabetes mellitus than in the controls
Kahwaji 2017	Serum	Jordan	T2DM	There were no significant differences in omentin levels between subjects with GDM and controls
Kocijancic 2015	Serum	Croatia	T2DM	There were no significant differences in omentin levels between subjects with GDM and controls
Lewandowski 2010	Serum	Poland	GDM	There were no significant differences in omentin levels between subjects with GDM and controls
Madsen 2015	Serum	Denmark	T2DM	Higher level of omentin-1 was observed in patients with diabetes mellitus than in the controls
Mierzyński 2018	Serum	Poland	GDM	Lower level of omentin-1 was observed in patients with diabetes mellitus than in the controls
Motawi 2017	Serum	Egypt	T2DM	Lower level of omentin-1 was observed in patients with diabetes mellitus than in the controls
Nurten 2018	Serum	Germany	T1DM	Higher level of omentin-1 was observed in patients with diabetes mellitus than in the controls
Pan 2010	Serum	China	T2DM	Lower level of omentin-1 was observed in patients with diabetes mellitus than in the controls
Polkowska 2016	Serum	Poland	T1DM	Lower level of omentin-1 was observed in patients with diabetes mellitus than in the controls
Tan 2008	Plasma	Poland	T1DM	Lower level of omentin-1 was observed in patients with diabetes mellitus than in the controls
Tekce 2014	Serum	Turkey	T2DM	Lower level of omentin-1 was observed in patients with diabetes mellitus than in the controls
Tsiotra 2018	Serum	Greece	GDM	Lower level of omentin-1 was observed in patients with diabetes mellitus than in the controls
Urbanova 2014	Serum	Czech	T2DM	Lower level of omentin-1 was observed in patients with diabetes mellitus than in the controls
Wan 2015	Serum	China	T2DM	Lower level of omentin-1 was observed in patients with diabetes mellitus than in the controls
Yan 2011A	Serum	China	T2DM	Lower level of omentin-1 was observed in patients with diabetes mellitus than in the controls
Yan 2011B	Plasma	China	T2DM	Lower level of omentin-1 was observed in patients with diabetes mellitus than in the controls
Yoo 2011	Serum	Korea	T2DM	Lower level of omentin-1 was observed in patients with diabetes mellitus than in the controls
Zhang 2014	Serum	China	T2DM	Lower level of omentin-1 was observed in patients with diabetes mellitus than in the controls
Abd El Dayem 2015	Serum	Egypt	T1DM	Lower level of omentin-1 was observed in patients with diabetes mellitus than in the controls
Nassif 2013	Serum	Egypt	T2DM	There was no significant difference in Omentin1 level between diabetic patients and control group.
Matloch 2018	Serum	Czech	T2DM	Lower level of omentin-1 was observed in patients with diabetes mellitus than in the controls
Gürsoy 2010	Plasma	Turkey	T2DM	Lower level of omentin-1 was observed in patients with diabetes mellitus than in the controls
Flehmig 2014	Serum	Germany	T2DM	Higher level of omentin-1 was observed in patients with diabetes mellitus than in the controls
Dogan 2016	Serum	Turkey	T2DM	Lower level of omentin-1 was observed in patients with diabetes mellitus than in the controls
Komosinska-vassev 2019	Plasma	Poland	T2DM	Higher level of omentin-1 was observed in patients with diabetes mellitus than in the controls
Rathwa 2019	Plasma	India	T2DM	Lower level of omentin-1 was observed in patients with diabetes mellitus than in the controls
Tuttolomondo 2019	Serum	Italy	T2DM	Lower level of omentin-1 was observed in patients with diabetes mellitus than in the controls
Souvannavong-vilivong 2019	Serum	Thailand	GDM	Lower level of omentin-1 was observed in patients with diabetes mellitus than in the controls
Aminilari 2017	Serum	Canada	T2DM	Combined exercise was efficient in increasing serum omentin-1 among women with T2DM
Arman 2017	Serum	Turkey	T2DM	In type 2 diabetes mellitus patients using insulin there was a significant decrease in Omentin-1 levels compared with the initial results
Arslan 2017	Serum	Turkey	T2DM	Diet plus metformin treatment decreased the omentin levels in type 2 diabetes patients
Biscetti 2019	Serum	Italy	T2DM	Omentin-1 is reduced in type 2 diabetic patients with peripheral artery disease and that omentin-1 levels are related to disease severity.
Esteghamati 2013	Serum	Iran	T2DM	After three months, metformin decreased omentin concentrations in T2DM
Kaushik 2018	Plasma	India	Prediabetic	Concentration of plasma omentin-1 decreased and insulin resistance increased in obese prediabetics compared to obese normoglycaemics and healthy controls.
Moreno-navarrete 2011	Serum	Spain	Impaired glucose tolerance	Concentration of omentin-1 decreased in impaired glucose tolerance.
Sperling 2016	Serum	Poland	Impaired glucose tolerance	Concentration of omentin-1 decreased in impaired glucose tolerance.
Lesná 2015	Serum	Czech Republic	T1DM	Omentin-1 plasma levels significantly increased during the weight reduction programme.

Characteristics of eligible studies are shown in each independent case. T1DM, type 1 diabetes mellitus; T2DM, type 2 diabetes mellitus; GDM, gestational diabetes mellitus.

**Table 2:** Characteristics of the studies included for the meta-analysis of omentin-1 concentrations in diabetes mellitus.

Study	Study design	Male, n(%)	BMI	Mean Age	Type	Methods	Frozen	NOS
Abd-elbaky 2015	Case-control	80(100%)	32.4±1.4	42.0±3.0	T2DM	ELISA	−80℃	7
Abdelraoufkorany 2018	Case-control	12(48%)	26.6±2.9	56.5±7.2	T2DM	ELISA	NR	6
Abell 2017	Case-control	25(0%)	28.0±2.6	32.7±3.3	GDM	ELISA	−80℃	7
Ahmed 2018	Case-control	40(100%)	28.8±6.8	57.6±15.6	T2DM	ELISA	−20℃	8
Akbarzadeh 2012	Case-control	41(46%)	27.6±4.3	40.5±9.6	T2DM	ELISA	−70℃	7
Akour 2016	Case-control	68(66%)	33.3±3.0	51.0±4.8	T2DM	ELISA	−80℃	8
Cai 2008	Case-control	35(51%)	28.5±2.7	48.8±11.7	T2DM	ELISA	−80℃	8
Dayem 2015	Case-control	62(100%)	24.9±4.2	16.3±1.5	T1DM	ELISA	NR	6
El-mesallamy 2011	Case-control	35(66%)	29.0±0.8	58.0±1.0	T2DM	ELISA	−80℃	7
Elsaid 2018	Case-control	60(0%)	39.4±7.0	49.5±6.0	T2DM	ELISA	−20℃	8
Franz 2018	Case-control	96(0%)	28.0±6.6	34.1±7.4	GDM	ELISA	−80℃	9
Greulich 2013	Case-control	78(100%)	28.9±1.1	71.9±2.4	T2DM	ELISA	−80℃	7
Hayashi 2018	Case-control	246(58%)	25.0±2.2	65.0±5.7	T2DM	ELISA	NR	5
Herder 2017	Case-control	34(52%)	27.7±4.0	68.6±4.9	T2DM	ELISA	NR	6
Kahwaji 2017	Cross sectional	35(34%)	33.5±5.9	51.9±10.9	T2DM	ELISA	−80℃	7
Kocijancic 2015	Case-control	57(34%)	25.6±3.7	70.0±3.0	T2DM	ELISA	NR	6
Lewandowski 2010	Case-control	20(100%)	30.3±3.1	29.7±3.7	GDM	ELISA	NR	6
Madsen 2015	Case-control	3(30%)	27.6±2.7	56.0±2.0	T2DM	ELISA	−80℃	7
Mierzyński 2018	Case-control	63(0%)	24.8±2.5	28.6±5.1	GDM	ELISA	−70℃	8
Motawi 2017	Case-control	23(51%)	23.1±1.8	53.7±7.6	T2DM	ELISA	NR	6
Nurten 2018	Case-control	115(47%)	23.9±9.2	13.0±3.9	T1DM	ELISA	−80℃	7
Pan 2010	Case-control	30(55%)	25.9±3.3	39.9±4.8	T2DM	ELISA	−70℃	7
Polkowska 2016	Case-control	5(50%)	18.3±5.7	10.4±2.8	T1DM	ELISA	NR	6
Tan 2008	Case-control	19(100%)	25.1±3.3	30.3±4.9	T1DM	ELISA	NR	6
Tekce 2014	Case-control	36(66%)	26.1±4.8	55.2±8.9	T2DM	ELISA	−80℃	8
Tsiotra 2018	Case-control	15(100%)	36.0±1.5	36.1±1.2	GDM	ELISA	−80℃	7
Urbanova 2014	Case-control	11(0%)	52.6±2.6	56.9±2.8	T2DM	ELISA	−80℃	6
Wan 2015	Case-control	60(100%)	23.8±3.0	58.7±9.0	T2DM	ELISA	−80℃	7
Yan 2011A	Case-control	18(51%)	24.3±0.6	55.5±1.7	T2DM	ELISA	−70℃	7
Yan 2011B	Case-control	13(43%)	24.4±3.3	53.1±7.7	T2DM	ELISA	−80℃	7
Yoo 2011	Case-control	10(33%)	23.9±2.3	53.1±6.8	T2DM	ELISA	−80℃	7
Zhang 2014	Case-control	20(57%)	32.4±5.5	65.1±10.7	T2DM	ELISA	−80℃	8
Abd El Dayem 2015	Case-control	31(50%)	24.9±4.2	16.3±1.5	T1DM	ELISA	NR	7
Nassif 2013	Case-control	19(87%)	24.3±0.3	48.9±1.5	T2DM	ELISA	−80℃	5
Matloch 2018	Case-control	7(78%)	30.4±3.59	67.0±8.76	T2DM	ELISA	−80℃	5
Gürsoy 2010	Case-control	0(0%)	30.5±4.8	52.8±10.7	T2DM	ELISA	−75℃	6
Flehmig 2014	Cross sectional	34(50%)	46.9±10.1	53.0±13.0	T2DM	ELISA	NR	7
Dogan 2016	Case-control	7(47%)	22.8±1.9	48.3±6.1	T2DM	ELISA	−80℃	5
Komosinska-vassev 2019	Case-control	23(55%)	33.1±4.1	61.0±5.6	T2DM	ELISA	−20℃	7
Rathwa 2019	Case-control	123(49%)	28.2±5.0	55.9±10.5	T2DM	ELISA	−20℃	7
Tuttolomondo 2019	Case-control	23(57%)	29.4±5.5	61.4±11.4	T2DM	ELISA	−80℃	6
Souvannavong-vilivong 2019	Case-control	0(0%)	29.0±5.0	38.2±1.3	GDM	ELISA	−70℃	6

Characteristics of eligible studies are shown in each independent case. NOS, Newcastle-Ottawa Scale; BMI, Body Mass Index; ELISA, enzyme linked immunosorbent assay; NR, not report; T1DM, type 1 diabetes mellitus; T2DM, type 2 diabetes mellitus; GDM, gestational diabetes mellitus.

### Overall comparison

**Fig 2:**
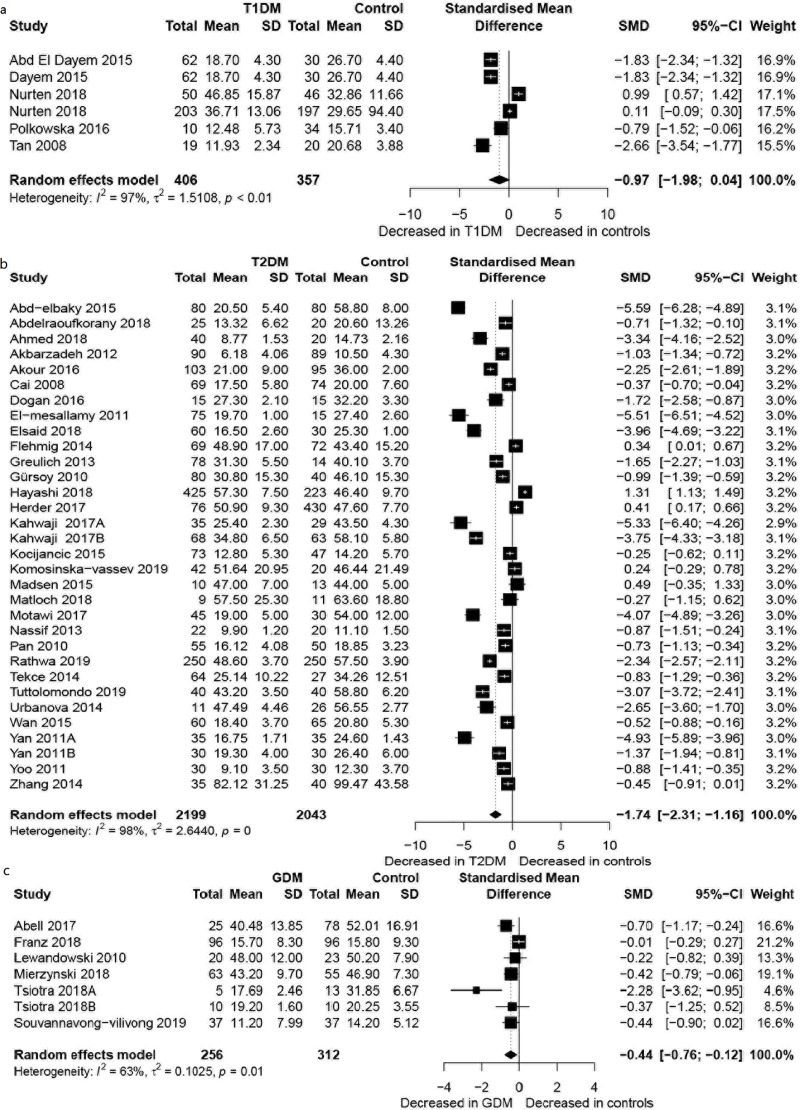
**Forest plot of gestational diabetes mellitus (a), type 1 diabetes mellitus (b) and type 2 diabetes mellitus (c).** Study effect sizes of omentin-1 concentration differences between diabetes mellitus and controls. Each data marker represents a study, and the size of the data marker is proportional to the total number of individuals in that study. The summary effect size for each omentin-1 concentration is denoted by a diamond. T1DM, type 1 diabetes mellitus; T2DM, type 2 diabetes mellitus; GDM, gestational diabetes mellitus; SMD, standardized mean difference.

Referring to the 6 studies that compared omentin-1 concentrations between 406 T1DM patients and 357 controls (Fig 2A), the concentrations were not significantly different between T1DM patients and controls, whereas for the 32 studies that compared omentin-1 concentrations between 2,199 T2DM patients and 2,043 controls (Fig 2B), omentin-1 concentrations were significantly decreased in the T2DM patients than in the controls (SMD = -1.74, 95% CI: -2.31; -1.16, p<0.001). Moreover, for the 7 studies that compared omentin-1 concentrations between 256 GDM patients and 312 controls (Fig 2C), results indicated that omentin-1 concentrations were significantly decreased in GDM patients, when compared with the controls (SMD = -0.44, 95% CI: -0.76; -0.12, *p =* 0.007), but the heterogeneity was moderate (*I^2^* = 63.20%).

### Subgroup analyses

Subgroup analyses results are presented in Table 3. Lower omentin-1 concentrations were observed in the GDM patients, unlike in the controls (SMD = -0.36, 95% CI: -0.67; -0.05, p = 0.022) for studies on patients with BMI of less than 30, and moderate heterogeneity was detected (I^2^ = 61.40%). Focusing on the serum omentin-1 concentrations, it was found that omentin-1 concentrations significantly decreased in the GDM patients than in the controls (SMD = -0.54, 95% CI: -0.85; -0.23, p<0.001), with lower heterogeneity (I^2^ = 43.20%). Similarly, lower omentin-1 concentrations were observed in the GDM patients, unlike in the controls (SMD = -0.47, 95% CI: -0.73; -0.21, *p*<0.001) for studies on patients aged less than 34 years, and no heterogeneity was detected (*I^2^* = 0%).

**Table 3:** Subgroup analysess of gestational diabetes mellitus, type 1 diabetes mellitus and type 2 diabetes mellitus.

	Number of studies	SMD (95% CI) Z P value		Heterogeneity	
			H(95%CI)	τ^2^	I^2^
**GDM**					
**All**	7	-0.4395 [-0.7567; -0.1223] -2.72 0.0066	1.65 [1.09; 2.48]	0.10	63.20%
**Material**					
**Serum**	6	-0.5386 [-0.8489; -0.2283] -3.40 0.0007	1.33 [1.00; 2.11]	0.06	43.20%
**BMI**					
**≤30**	4	-0.3617 [-0.6709; -0.0525] -2.29 0.0218	1.61 [1.00; 2.78]	0.06	61.40%
**>30**	3	-0.8041 [-1.8345; 0.2263] -1.53 0.1261	1.98 [1.08; 3.61]	0.60	74.40%
**Mean Age**					
**≤34**	3	-0.4740 [-0.7327; -0.2153] -3.59 0.0003	1.00	0.00	0.00%
**>34**	4	-0.5216 [-1.1268; 0.0837] -1.69 0.0912	2.02 [1.22; 3.36]	0.25	75.60%
**T1DM**					
**All**	6	-0.9702 [-1.9816; 0.0411] -1.88 0.0601	5.46 [4.32; 6.89]	1.51	96.60%
**Material**					
**Serum**	5	-0.6581 [-1.6847; 0.3686] -1.26 0.2090	5.47 [4.22; 7.09]	1.31	96.70%
**T2DM**					
**All**	32	-1.7352 [-2.3094; -1.1609] -5.92 < 0.0001	7.46 [6.90; 8.06]	2.64	98.20%
**Material**					
**Serum**	26	-1.8684 [-2.5560; -1.1809] -5.33 < 0.0001	7.65 [7.03; 8.33]	3.09	98.30%
**Plasma**	6	-1.1994 [-1.9451; -0.4536] -3.15 0.0016	4.64 [3.59; 6.01]	0.81	95.40%
**BMI**					
**≤30**	20	-1.2941 [-1.9482; -0.6400] -3.88 0.0001	7.33 [6.63; 8.10]	2.14	98.10%
**>30**	12	-2.3573 [-3.4000; -1.3145] -4.43 < 0.0001	6.87 [6.03; 7.84]	3.55	97.90%
**Frozen**					
**Yes**	26	-2.0223 [-2.5496; -1.4949] -7.52 < 0.0001	5.37 [4.83; 5.97]	1.77	96.50%
**No**	6	-0.4244 [-1.3280; 0.4792] -0.92 0.3573	6.80 [5.55; 8.33]	1.22	97.80%

Subgroup analyses were performed to compare the concentration of each omentin-1 between the DM and the controls. Heterogeneity was quantified using I^2^. SMD, standardized mean difference; T1DM, type 1 diabetes mellitus; T2DM, type 2 diabetes mellitus; GDM, gestational diabetes mellitus.

Furthermore, significantly lower serum omentin-1 concentrations were observed in the T2DM patients than controls (SMD = -1.87, 95% CI: -2.56; -1.18, p<0.001), and significantly lower plasma omentin-1 concentrations were observed in the T2DM patients than controls (SMD = -1.20, 95% CI: -1.95; -0.45, p = 0.002). Additionally, significantly lower omentin-1 concentrations were observed in T2DM patients than controls (SMD = -2.02, 95% CI: -2.55; -1.49, p<0.001), when samples were stored and transported refrigerated.

It was also found that different sample types did not change the results in serum. Apparently, due to the small number of studies on plasma samples, subgroup analysis in GDM and T1DM was not possible to be performed. However, in GDM, part of the heterogeneity could be explained, which might suggest that although omentin-1 concentrations had a downward trend in both serum and plasma, the degree might be different.

### Sensitivity and bias analysis

In sensitivity analysis, it was observed that any single study or cluster of studies with some common attributes had minimal influence on the SMD and corresponding 95% CI. Also, the total number of studies reporting omentin-1 concentrations for GDM and T1DM was less than 10 for each comparison, thus publication bias was not reported for these groups. Although it was hard to observe asymmetry in the shape of the funnel plot for T2DM, the Egger’s test was significant (*p*<0.001), implying that there may be publication bias for this comparison.

## Discussion

Omentin-1 concentrations were lower in T2DM and GDM subjects unlike in the controls. However, there was remarkably high heterogeneity. Also, there was a strong possibility of publication bias. This was not a surprise given the large variations in the study populations and regions [17]. Thus, caution should be taken when interpreting these results. Nonetheless, these data are consistent with the hypothesis that dyssecretosis of the omentin-1 has a relationship with the pathophysiology of T2DM and GDM.

No difference in omentin-1 concentrations was found between T1DM patients and controls. However, the number of patients with TIDM was the smallest among the three subgroups of DM. As a result, there is need for further investigations to ascertain this association in T1DM. Up to now, a number of factors have been proposed to cause metabolic disorder in DM patients, including β-cell dysfunction, insulin resistance, and bodyweight gain, or a combination of these [22]. While T1DM is common in children and young people, and is related to an autoimmune process destroying pancreatic beta cells, T2DM and GDM have been shown to be associated with obesity and endocrine activity of adipose tissue [2, 23]. Omentin-1 may be essential in maintaining energy balance during pathogenesis of insulin resistance and T2DM even if the mechanisms are not fully understood [24]. Findings of previous experimental and clinical studies were in consistent with the hypothesis that the omentin-1 concentrations affect the pathophysiology of diabetes.

**Fig 3:**
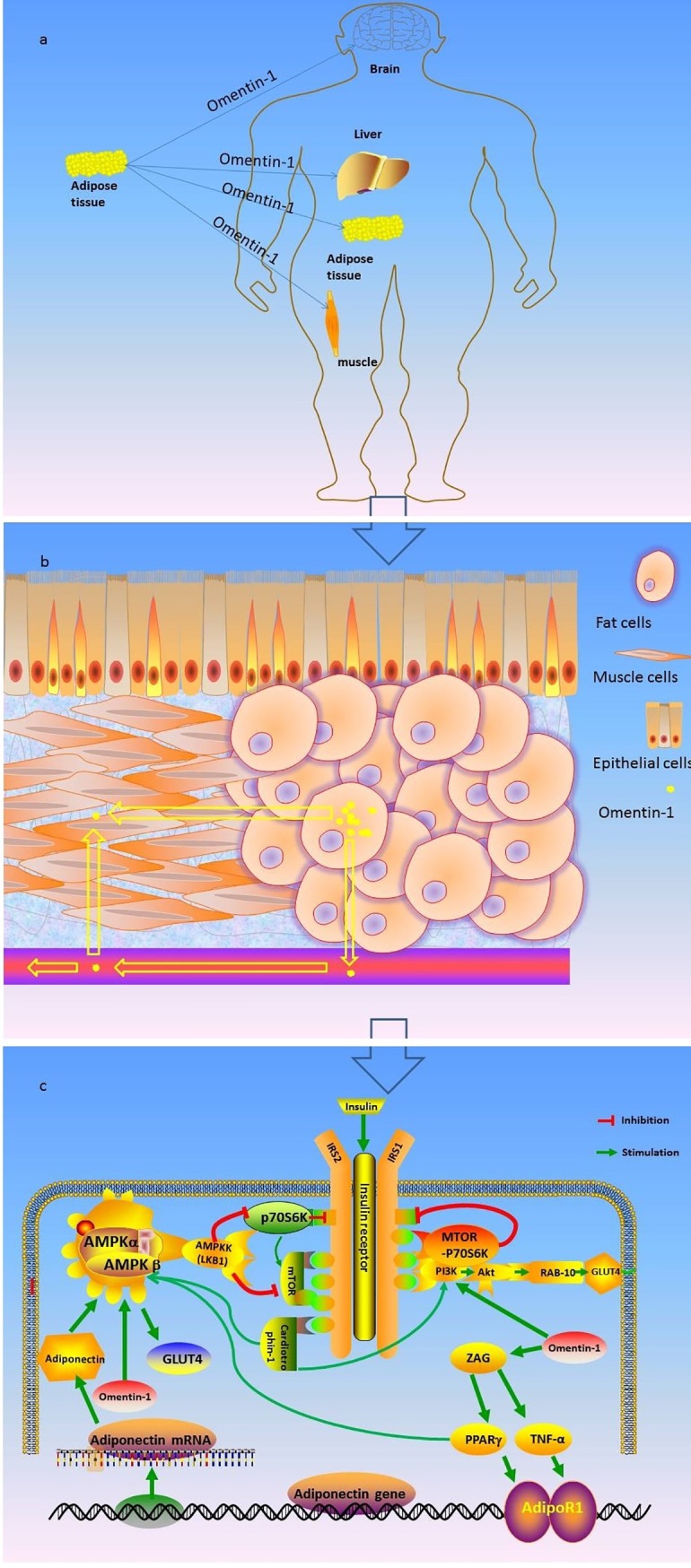
Summarizes the hypothesis mechanism process of omentin-1 on insulin signaling cascade. Fig 3 Schematic representation of the effects of omentin-1 on insulin signaling cascade that explain positive effects of these adipokines on glycemic control. Akt: protein kinase b; TNF-α: Tumor Necrosis Factor; AMPK: AMP activated protein-kinase; ZAG: Zinc-α2-glycoprotein; AS160: Akt substrate of 160 kDa; GLUT: glucose transporter; IRS: insulin receptor substrate; mTOR: mammalian target of rapamicin; PPAR-γ: peroxisome proliferator-activated receptor; AdipoR1: Adipo receptor1; PI3K: phosphatidylinositol 3-kinase; RAB-10: Ras-related protein RAB-10. Fig 1 is drawn by the KA, without any copyright disputes.

As regards direct hypothetical mechanisms shown in Fig 3 (drawn by the KA), omentin-1 modulates the insulin sensitivity and secretion and this affects organs such as adipose, muscle, brain, liver, and other tissues (Fig 3A and Fig 3B). Likewise, some experimental studies have revealed that omentin-1 stimulates insulin receptor substrate (IRS) by restraining the rapamicin (mTOR-p70S6K), which subsequently is a result of Adenosine 5-monophosphate (AMP)-activated protein kinase (AMPK) activation [25]. Also, omentin-1 up regulates adiponectin gene expression and Zinc-alpha2-glycoprotein (ZAG) mRNA expression, which may directly stimulate lipolysis through interaction with AMPK [26]. For instance, omentin-1 can enhance the effect of insulin by increasing glucose uptake by visceral fat adipose tissue in vitro via Akt signaling, mediated by insulin [27]. Fig 3C shows the hypothesis mechanism process. Furthermore, it is well known that indirect hypothetical mechanisms are mainly related to omentin-1, which is influential in adipose tissue accumulation, inflammation, and adverse distribution of fat. These then contribute to affect glucose metabolism. For example, preclinical evidence has shown that omentin-1 decreased in hyperglycemia subjects, and was inversely associated with chronic inflammation [28].

Interestingly, it was noted that several studies have reported the effects of clinical interventions on omentin-1 concentrations in diabetic patients. For instance, Aminilari et al found that exercising can increase the serum omentin-1 concentration of T2DM [29], while in other studies, metformin treatment intervention could reduce the serum omentin-1 concentration of T2DM [30, 31]. This seems to suggest that aerobic exercises could be used to improve levels of omentin-1 in diabetic patients, whereas drug interventions have had an inhibitory effect. Meanwhile, it was noticed that some studies reported that concentrations of omentin-1 decreased in pre-diabetes[32]. The possible explanation for this phenomenon is that, as omentin-1 could increases insulin sensitivity, its decrease may be the cause of impaired glucose homeostasis in prediabetic patients [33].

For example, experimental studies indicated that omentin-1 could regulate lipid metabolism and balancing of energy presumably through activation of AMPK pathways in cell models [25]. It is well known that AMPK plays a major role as an energy sensor in regulating energy balance. It produces energy by glucose uptake and activates energy metabolism. It is now widely accepted that AMPK inhibits other metabolic process that consume energy through protein synthesis gluconeogenesis, and lipogenesis mechanisms [34]. In addition, the omentin-1 was found to increase insulin induction of Akt/PKB phosphorylation in isolated adipocytes and to enhance glucose uptake [35]. On the other hand, in clinical studies, circulating omentin-1 concentrations were decreased in people with obesity/diabetes. In obesity, concentrations of omentin-1 were decreased in plasma and adipose tissue. Besides, omentin-1 concentrations were found to be positively correlated with high-density lipoprotein and adiponectin and, in contrast, they were negatively correlated with body mass index and insulin resistance [36].

Therefore, it may be suggested that insulin and glucose administration to visceral fat adipose tissue could result in dose-dependent reductions of omentin-1 expression.

This study has made the following improvements on the meta-analysis by As'habi et al. First, this study used a wide range of databases to retrieve 51 eligible studies, which is an improvement on the previous meta-analysis, which used only 28 articles. Second, the GDM subgroup which was not included in the previous meta-analysis was included in this study and yielded significant results. Finally, unlike in the previous meta-analysis, this study further elaborated on the omentin-1 possible mechanism in the pathophysiology of diabetes [14].

### Subgroup analyses

Heterogeneity was significantly high in the assessment of concentrations of omentin-1 in relation to DM, indicating that some unmeasured or unassessed factors might have been responsible. As regards GDM, results of subgroup analyses showed that patient's age and BMI contributed significantly to the heterogeneity, and without significant residual heterogeneity. Thus, it may be deduced that homogenous groups may be formed by different countries or regions when exploring the relationship between omentin-1 and GDM [37]. In addition, a number of studies confirmed that advanced maternal age was a risk factor for GDM [38]. Likewise, clinical evidence has shown that women in advanced maternal age were more likely to be overweight/obese, and lower serum omentin-1 were prevalent in morbidly obese women than in normal weight women, and its concentration inversely correlated with glucose metabolism markers [39]. Thus, it might suggest that age-mediated output of omentin-1 in adipose tissue was affected in GDM. Results of this study also suggest that GDM in women in advanced maternal age may be related to omentin-1 secretion disorder.

Additionally, with respect to T2DM, subgroup analyses indicated that neither storing nor transporting samples in cold storage had a significant impact on the results, which indicated that the concentration of omentin-1 was unstable in such samples. Therefore, future studies may need to store and transport serum or plasma samples in cold storage [13].

### Study limitations

In spite of this study showing an association between omentin-1 concentrations and some types of DM, some limitations should be acknowledged. First, there was a small number of eligible studies on the association between T1DM and concentrations of omentin-1, which may lead to lack of power in this analysis. Second, given high heterogeneity and publication bias among eligible studies, comparability among studies may have been compromised. Although some subgroup analyses explained the source of heterogeneity, other factors that could interact with concentrations of omentin-1, such as physical activity, cigarettes smoking, alcohol consumption, and blood pressure were not taken into account in the original studies. Finally, all eligible studies used either case control or cross-sectional design, which cannot make causality inference.

## Conclusion

Despite the preceding limitations, this study managed to provide significant evidence to suggest that there is a relationship between omentin-1 concentrations and GDM/T2DM. Specifically, significantly lower omentin-1 concentrations were observed in people with GDM or T2DM than in the controls, whereas no difference of the same was found between the T1DM and controls. Although the exact role of omentin-1 remains to be defined in glucose metabolism and its target tissues; and in receptors and modes of action through which it signals, the foregoing findings suggest new perspectives in early diagnosis, identification of novel biomarkers, and providing novel targets for pharmacological interventions.

## Supporting information

S1 Table Search strategies. Details of search strategy.
(DOC)

S2 Table PRISMA checklist. (DOC)

S3 Table References for included studies. (DOC)

S4 Table Details of quality evaluation included in the study. (DOC)

## References

[1] Bohula EA, Scirica BM, Inzucchi SE, McGuire DK, Keech AC, Smith SR, et al Effect of lorcaserin on prevention and remission of type 2 diabetes in overweight and obese patients (CAMELLIA-TIMI 61): a randomised, placebo-controlled trial. Lancet (London, England). 2018;392(10161):2269–79. Epub 2018/10/09. 10.1016/s0140-6736(18)32328-6 . 30293771

[2] DiMeglio LA, Evans-Molina C, Oram RA. Type 1 diabetes. Lancet (London, England). 2018;391(10138):2449–62. Epub 2018/06/20. 10.1016/s0140-6736(18)31320-5 . 29916386 PMC6661119

[3] Bradshaw JM, Ensor SJA, Lorenz HAL. Gestational Diabetes and Childhood Obesity. Jama. 2019;321(7):708 Epub 2019/02/20. 10.1001/jama.2018.19750 . 30778593

[4] Nathan DM. Diabetes: Advances in Diagnosis and Treatment. Jama. 2015;314(10):1052–62. Epub 2015/09/09. 10.1001/jama.2015.9536 . 26348754

[5] Lotta LA, Wittemans LBL, Zuber V, Stewart ID, Sharp SJ, Luan J, et al Association of Genetic Variants Related to Gluteofemoral vs Abdominal Fat Distribution With Type 2 Diabetes, Coronary Disease, and Cardiovascular Risk Factors. Jama. 2018;320(24):2553–63. Epub 2018/12/24. 10.1001/jama.2018.19329 . 30575882 PMC6583513

[6] Li S, Shin HJ, Ding EL, van Dam RM. Adiponectin levels and risk of type 2 diabetes: a systematic review and meta-analysis. Jama. 2009;302(2):179–88. Epub 2009/07/09. 10.1001/jama.2009.976 . 19584347

[7] Subiabre M, Villalobos-Labra R, Silva L, Fuentes G, Toledo F, Sobrevia L. Role of insulin, adenosine, and adipokine receptors in the foetoplacental vascular dysfunction in gestational diabetes mellitus. Biochimica et biophysica acta Molecular basis of disease. 2019 Epub 2019/01/21. 10.1016/j.bbadis.2018.12.021 . 30660686

[8] Vedal TSJ, Steen NE, Birkeland KI, Dieset I, Reponen EJ, Laskemoen JF, et al Adipokine levels are associated with insulin resistance in antipsychotics users independently of BMI. Psychoneuroendocrinology. 2019;103:87–95. Epub 2019/01/20. 10.1016/j.psyneuen.2019.01.001 . 30659986

[9] Greulich S, Chen WJ, Maxhera B, Rijzewijk LJ, van der Meer RW, Jonker JT, et al Cardioprotective properties of omentin-1 in type 2 diabetes: evidence from clinical and in vitro studies. PLoS One. 2013;8(3):e59697 Epub 2013/04/05. 10.1371/journal.pone.0059697 23555749 PMC3612072

[10] Yoo HJ, Hwang SY, Hong HC, Choi HY, Yang SJ, Seo JA, et al Association of circulating omentin-1 level with arterial stiffness and carotid plaque in type 2 diabetes. Cardiovasc Diabetol. 2011;10:103 Epub 2011/11/24. 10.1186/1475-2840-10-103 22108456 PMC3235986

[11] Yan P, Li L, Yang M, Liu D, Liu H, Boden G, et al Effects of the long-acting human glucagon-like peptide-1 analog liraglutide on plasma omentin-1 levels in patients with type 2 diabetes mellitus. Diabetes Res Clin Pract. 2011;92(3):368–74. Epub 2011/04/05. 10.1016/j.diabres.2011.02.030 . 21458097

[12] Abdelraouf Korany M, Sonbol A, Mohamed Elgouhary S. Omentin-1 and diabetic retinopathy in type 2 diabetic patients. Alexandria Journal of Medicine. 2018;54(4):323–6. 10.1016/j.ajme.2018.04.003

[13] Hayashi M, Morioka T, Hatamori M, Kakutani Y, Yamazaki Y, Kurajoh M, et al Plasma omentin levels are associated with vascular endothelial function in patients with type 2 diabetes at elevated cardiovascular risk. Diabetes Res Clin Pract. 2019;148:160–8. Epub 2019/01/15. 10.1016/j.diabres.2019.01.009 . 30641171

[14] As´habi A, Sadeghi M, Arab A, Hajianfar H. The association between omentin and diabetes: a systematic review and meta-analysis of observational studies. Diabetes, Metabolic Syndrome and Obesity: Targets and Therapy. 2019;Volume 12:1277–86. 10.2147/DMSO.S206981 31447571 PMC6683169

[15] Moher D, Liberati A, Tetzlaff J, Altman DG. Preferred reporting items for systematic reviews and meta-analyses: the PRISMA statement. BMJ (Clinical research ed). 2009;339:b2535 Epub 2009/07/23. 10.1136/bmj.b2535 19622551 PMC2714657

[16] Stang A. Critical evaluation of the Newcastle-Ottawa scale for the assessment of the quality of nonrandomized studies in meta-analyses. Eur J Epidemiol. 2010;25(9):603–5. Epub 2010/07/24. 10.1007/s10654-010-9491-z . 20652370

[17] Higgins JP, Thompson SG, Deeks JJ, Altman DG. Measuring inconsistency in meta-analyses. BMJ (Clinical research ed). 2003;327(7414):557–60. Epub 2003/09/06. 10.1136/bmj.327.7414.557 12958120 PMC192859

[18] DerSimonian R, Laird N. Meta-analysis in clinical trials revisited. Contemp Clin Trials. 2015;45(Pt A):139–45. Epub 2015/09/08. 10.1016/j.cct.2015.09.002 26343745 PMC4639420

[19] Higgins JP, Thompson SG. Quantifying heterogeneity in a meta-analysis. Statistics in medicine. 2002;21(11):1539–58. Epub 2002/07/12. 10.1002/sim.1186 . 12111919

[20] Pan X, Wang Z, Wu X, Wen SW, Liu A. Salivary cortisol in post-traumatic stress disorder: a systematic review and meta-analysis. BMC Psychiatry. 2018;18(1):324 Epub 2018/10/07. 10.1186/s12888-018-1910-9 30290789 PMC6173866

[21] Egger M, Davey Smith G, Schneider M, Minder C. Bias in meta-analysis detected by a simple, graphical test. BMJ (Clinical research ed). 1997;315(7109):629–34. Epub 1997/10/06. 10.1136/bmj.315.7109.629 9310563 PMC2127453

[22] Hernandez AF, Green JB, Janmohamed S, D'Agostino RB Sr., Granger CB, Jones NP, et al Albiglutide and cardiovascular outcomes in patients with type 2 diabetes and cardiovascular disease (Harmony Outcomes): a double-blind, randomised placebo-controlled trial. Lancet (London, England). 2018;392(10157):1519–29. Epub 2018/10/07. 10.1016/s0140-6736(18)32261-x . 30291013

[23] Ambery P, Parker VE, Stumvoll M, Posch MG, Heise T, Plum-Moerschel L, et al MEDI0382, a GLP-1 and glucagon receptor dual agonist, in obese or overweight patients with type 2 diabetes: a randomised, controlled, double-blind, ascending dose and phase 2a study. Lancet (London, England). 2018;391(10140):2607–18. Epub 2018/06/28. 10.1016/s0140-6736(18)30726-8 . 29945727

[24] Elsaid NH, Sadik NA, Ahmed NR, Fayez SE, Mohammed NAE. Serum omentin-1 levels in type 2 diabetic obese women in relation to glycemic control, insulin resistance and metabolic parameters. J Clin Transl Endocrinol. 2018;13:14–9. Epub 2018/07/20. 10.1016/j.jcte.2018.05.003 30023310 PMC6047309

[25] Hernandez-Diaz A, Arana-Martinez JC, Carbo R, Espinosa-Cervantes R, Sanchez-Munoz F. [Omentin: Role in insulin resistance, inflammation and cardiovascular protection]. Archivos de cardiologia de Mexico. 2016;86(3):233–43. Epub 2016/01/19. 10.1016/j.acmx.2015.09.010 . 26778502

[26] Ceperuelo-Mallafre V, Naf S, Escote X, Caubet E, Gomez JM, Miranda M, et al Circulating and adipose tissue gene expression of zinc-alpha2-glycoprotein in obesity: its relationship with adipokine and lipolytic gene markers in subcutaneous and visceral fat. J Clin Endocrinol Metab. 2009;94(12):5062–9. Epub 2009/10/23. 10.1210/jc.2009-0764 . 19846741

[27] Yang RZ, Lee MJ, Hu H, Pray J, Wu HB, Hansen BC, et al Identification of omentin as a novel depot-specific adipokine in human adipose tissue: possible role in modulating insulin action. American journal of physiology Endocrinology and metabolism. 2006;290(6):E1253–61. Epub 2006/03/15. 10.1152/ajpendo.00572.2004 . 16531507

[28] Sengul E, Duygulu G, Dindar S, Bunul F. Serum omentin-1, inflammation and carotid atherosclerosis in patients with non-diabetic chronic kidney disease. Ren Fail. 2013;35(8):1089–93. Epub 2013/07/26. 10.3109/0886022X.2013.817256 . 23883412

[29] AminiLari Z, Fararouei M, Amanat S, Sinaei E, Dianatinasab S, AminiLari M, et al The Effect of 12 Weeks Aerobic, Resistance, and Combined Exercises on Omentin-1 Levels and Insulin Resistance among Type 2 Diabetic Middle-Aged Women. Diabetes Metab J. 2017;41(3):205–12. Epub 2017/05/26. 10.4093/dmj.2017.41.3.205 28537059 PMC5489501

[30] Esteghamati A, Noshad S, Rabizadeh S, Ghavami M, Zandieh A, Nakhjavani M. Comparative effects of metformin and pioglitazone on omentin and leptin concentrations in patients with newly diagnosed diabetes: a randomized clinical trial. Regul Pept. 2013;182:1–6. Epub 2013/01/19. 10.1016/j.regpep.2012.12.005 . 23328000

[31] Arslan I. Comparative effectiveness of diet alone and diet plus metformin treatment on omentin levels in type 2 diabetes patients with nonalcoholic fatty liver disease: a prospective randomized trial. Periodicum Biologorum. 2017;119(1):9–15. 10.18054/pb.v119i1.4180

[32] Kaushik N, Kaushik R, Dixit P, Tyagi M, Gambhir J, Madhu SV, et al Plasma Omentin-1 Level and its Relationship with Insulin Resistance in Obese Prediabetics. JOURNAL OF CLINICAL AND DIAGNOSTIC RESEARCH. 2018;12 10.7860/JCDR/2018/31845.11432

[33] Moreno-Navarrete JM, Ortega F, Castro A, Sabater M, Ricart W, Fernandez-Real JM. Circulating omentin as a novel biomarker of endothelial dysfunction. Obesity (Silver Spring, Md). 2011;19(8):1552–9. Epub 2011/02/05. 10.1038/oby.2010.351 . 21293447

[34] Castano D, Larequi E, Belza I, Astudillo AM, Martinez-Anso E, Balsinde J, et al Cardiotrophin-1 eliminates hepatic steatosis in obese mice by mechanisms involving AMPK activation. J Hepatol. 2014;60(5):1017–25. Epub 2013/12/24. 10.1016/j.jhep.2013.12.012 . 24362075

[35] Pan HY, Guo L, Li Q. Changes of serum omentin-1 levels in normal subjects and in patients with impaired glucose regulation and with newly diagnosed and untreated type 2 diabetes. Diabetes Res Clin Pract. 2010;88(1):29–33. Epub 2010/02/05. 10.1016/j.diabres.2010.01.013 . 20129687

[36] de Souza Batista CM, Yang RZ, Lee MJ, Glynn NM, Yu DZ, Pray J, et al Omentin plasma levels and gene expression are decreased in obesity. Diabetes. 2007;56(6):1655–61. Epub 2007/03/03. 10.2337/db06-1506 . 17329619

[37] Pan X, Kaminga AC, Wen SW, Liu A. Catecholamines in Post-traumatic Stress Disorder: A Systematic Review and Meta-Analysis. Front Mol Neurosci. 2018;11:450 Epub 2018/12/20. 10.3389/fnmol.2018.00450 30564100 PMC6288600

[38] Hanprasertpong T, Kor-Anantakul O, Suwanrath C, Suntharasaj T, Pruksanusak N, Hanprasertpong J, et al Subsequent gestational diabetes mellitus prediction in advanced maternal age using amniotic fluid glucose concentration during second trimester genetic amniocentesis. Journal of obstetrics and gynaecology: the journal of the Institute of Obstetrics and Gynaecology. 2016;36(6):744–7. Epub 2016/03/29. 10.3109/01443615.2016.1150261 . 27018498

[39] Fasshauer M, Blüher M, Stumvoll M. Adipokines in gestational diabetes. The Lancet Diabetes & Endocrinology. 2014;2(6):488–99. 10.1016/s2213-8587(13)70176-1.</References> 24731659

