# Supplementary material for: Omentin-1 in diabetes mellitus: A systematic review and meta-analysis
Source: PLoS One. 2019 Dec 10;14(12):e0226292. doi: 10.1371/journal.pone.0226292 (PMC6903756; doi:10.1371/journal.pone.0226292)
Supplement: S1 Table — Details of search strategy. (DOC) [file pone.0226292.s001.doc]

Search strategies: details of search strategy.

((Diabetes Mellitus OR Diabetes OR Mellitus OR Diabetic OR Diabete OR Glycuresis OR Diabetics OR Type 2 diabetes OR T2D OR Type 2 diabetes mellitus OR T2DM OR Insulin-dependent diabetes mellitus OR IDDM OR Juvenile diabetes OR Non insulin-dependent diabetes mellitus OR NIDDM OR Adult-onset diabetes OR Type 1 diabetes OR T1D OR type 1 diabetes mellitus OR T1DM OR Gestational diabetes OR Gestational diabetes mellitus OR GDM)) AND (Omentin OR ITLN1 protein OR Intelectin OR Omentin-1 OR Intelectin-1)

196 of PubMed

TOPIC: (Diabetes Mellitus OR Diabetes OR Mellitus OR Diabetic OR Diabete OR Glycuresis OR Diabetics OR Type 2 diabetes OR T2D OR Type 2 diabetes mellitus OR T2DM OR Insulin-dependent diabetes mellitus OR IDDM OR Juvenile diabetes OR Non insulin-dependent diabetes mellitus OR NIDDM OR Adult-onset diabetes OR Type 1 diabetes OR T1D OR type 1 diabetes mellitus OR T1DM OR Gestational diabetes OR Gestational diabetes mellitus OR GDM) AND TOPIC: (Omentin OR ITLN1 protein OR Intelectin OR Omentin-1 OR Intelectin-1)

288 of Web of Science

('diabetes mellitus':ab,ti OR 'diabetes':ab,ti OR 'mellitus':ab,ti OR 'diabetic':ab,ti OR 'diabete':ab,ti OR 'glycuresis':ab,ti OR 'diabetics':ab,ti OR 'type 2 diabetes':ab,ti OR 't2d':ab,ti OR 'type 2 diabetes mellitus':ab,ti OR 't2dm':ab,ti OR 'insulin-dependent diabetes mellitus':ab,ti OR 'iddm':ab,ti OR 'juvenile diabetes':ab,ti OR 'non insulin-dependent diabetes mellitus':ab,ti OR 'niddm':ab,ti OR 'adult-onset diabetes':ab,ti OR 'type 1 diabetes':ab,ti OR 't1d':ab,ti OR 'type 1 diabetes mellitus':ab,ti OR 't1dm':ab,ti OR 'gestational diabetes':ab,ti OR 'gestational diabetes mellitus':ab,ti OR 'gdm':ab,ti) AND ('omentin':ab,ti OR 'itln1 protein':ab,ti OR 'intelectin':ab,ti OR 'omentin-1':ab,ti OR 'intelectin-1':ab,ti)

275 of Embase

Omentin OR ITLN1 protein OR Intelectin OR Omentin-1 OR Intelectin-1 in Title Abstract Keyword AND Diabetes Mellitus OR Diabetes OR Mellitus OR Diabetic OR Diabete OR Glycuresis OR Diabetics OR Type 2 diabetes OR T2D OR Type 2 diabetes mellitus OR T2DM OR Insulin-dependent diabetes mellitus OR IDDM OR Juvenile diabetes OR Non insulin-dependent diabetes mellitus OR NIDDM OR Adult-onset diabetes OR Type 1 diabetes OR T1D OR type 1 diabetes mellitus OR T1DM OR Gestational diabetes OR Gestational diabetes mellitus OR GDM in Title Abstract Keyword

32 of Cochrane

intitle:("Omentin-1" OR "Intelectin-1") AND intitle:("Diabetes Mellitus" OR "Diabetes" OR "Mellitus" OR "Diabetic" OR "diabetes" OR "glycoresins" OR "Diabetics")

629 of Google Scholar

TITLE-ABS-KEY ( diabetes AND mellitus OR diabetes OR mellitus OR diabetic OR diabete OR glycuresis OR diabetics OR type 2 diabetes OR t2d OR type 2 diabetes AND mellitus OR t2dm OR type 1 diabetes OR t1d OR type 1 diabetes AND mellitus OR t1dm OR gestational AND diabetes ) AND ALL ( omentin OR itln1 AND protein OR intelectin OR omentin-1 OR intelectin-1 )

197 of SCOPUS
