# Supplementary material for: Omentin-1 in diabetes mellitus: A systematic review and meta-analysis
Source: PLoS One. 2019 Dec 10;14(12):e0226292. doi: 10.1371/journal.pone.0226292 (PMC6903756; doi:10.1371/journal.pone.0226292)
Supplement: S3 Table — (DOC) [file pone.0226292.s003.doc]

**References for included studies**

| Study |  |  | Material | Country | Type | Outcome |
| --- | --- | --- | --- | --- | --- | --- |
| Abd-elbaky 2015 | | [1] | Serum | Egypt | T2DM | Lower level of omentin-1 was observed in patients with diabetes mellitus than in the controls |
| Abdelraoufkorany 2018[2] | | | Serum | Egypt | T2DM | Lower level of omentin-1 was observed in patients with diabetes mellitus than in the controls |
| Abell 2017 | | [3] | Serum | Australia | GDM | Lower level of omentin-1 was observed in patients with diabetes mellitus than in the controls |
| Ahmed 2018 | | [4] | Serum | Egypt | T2DM | Lower level of omentin-1 was observed in patients with diabetes mellitus than in the controls |
| Akbarzadeh 2012 | | [5] | Plasma | Iran | T2DM | Lower level of omentin-1 was observed in patients with diabetes mellitus than in the controls |
| Akour 2016 | | [6] | Serum | Jordan | T2DM | Lower level of omentin-1 was observed in patients with diabetes mellitus than in the controls |
| Cai 2008 |  | [7] | Serum | China | T2DM | Lower level of omentin-1 was observed in patients with diabetes mellitus than in the controls |
| Dayem 2015 | | [8] | Serum | Egypt | T1DM | Lower level of omentin-1 was observed in patients with diabetes mellitus than in the controls |
| El-mesallamy 2011[9] | | | Serum | Egypt | T2DM | There was no significant difference in Omentin1 level between diabetic patients and control group. |
| Elsaid 2018 | | [10] | Serum | Egypt | T2DM | Lower level of omentin-1 was observed in patients with diabetes mellitus than in the controls |
| Franz 2018 | | [11] | Plasma | Austria | GDM | There was no significant difference in Omentin1 level between diabetic patients and control group. |
| Greulich 2013 | | [12] | Plasma | Netherlands | T2DM | Lower level of omentin-1 was observed in patients with diabetes mellitus than in the controls |
| Hayashi 2018 | | [13] | Serum | Japan | T2DM | Higher level of omentin-1 was observed in patients with diabetes mellitus than in the controls |
| Herder 2017 | | [14] | Serum | Germany | T2DM | Higher level of omentin-1 was observed in patients with diabetes mellitus than in the controls |
| Kahwaji 2017 | | [15] | Serum | Jordan | T2DM | There were no significant differences in omentin levels between subjects with GDM and controls |
| Kocijancic 2015 | | [16] | Serum | Croatia | T2DM | There were no significant differences in omentin levels between subjects with GDM and controls |
| Lewandowski 2010 | | [17] | Serum | Poland | GDM | There were no significant differences in omentin levels between subjects with GDM and controls |
| Madsen 2015 | | [18] | Serum | Denmark | T2DM | Higher level of omentin-1 was observed in patients with diabetes mellitus than in the controls |
| Mierzyński 2018 | | [19] | Serum | Poland | GDM | Lower level of omentin-1 was observed in patients with diabetes mellitus than in the controls |
| Motawi 2017 | | [20] | Serum | Egypt | T2DM | Lower level of omentin-1 was observed in patients with diabetes mellitus than in the controls |
| Nurten 2018 | | [21] | Serum | Germany | T1DM | Higher level of omentin-1 was observed in patients with diabetes mellitus than in the controls |
| Pan 2010 |  | [22] | Serum | China | T2DM | Lower level of omentin-1 was observed in patients with diabetes mellitus than in the controls |
| Polkowska 2016 | | [23] | Serum | Poland | T1DM | Lower level of omentin-1 was observed in patients with diabetes mellitus than in the controls |
| Tan 2008 |  | [24] | Plasma | Poland | T1DM | Lower level of omentin-1 was observed in patients with diabetes mellitus than in the controls |
| Tekce 2014 | | [25] | Serum | Turkey | T2DM | Lower level of omentin-1 was observed in patients with diabetes mellitus than in the controls |
| Tsiotra 2018 | | [26] | Serum | Greece | GDM | Lower level of omentin-1 was observed in patients with diabetes mellitus than in the controls |
| Urbanova 2014 | | [27] | Serum | Czech | T2DM | Lower level of omentin-1 was observed in patients with diabetes mellitus than in the controls |
| Wan 2015 |  | [28] | Serum | China | T2DM | Lower level of omentin-1 was observed in patients with diabetes mellitus than in the controls |
| Yan 2011A | | [29] | Serum | China | T2DM | Lower level of omentin-1 was observed in patients with diabetes mellitus than in the controls |
| Yan 2011B | | [30] | Plasma | China | T2DM | Lower level of omentin-1 was observed in patients with diabetes mellitus than in the controls |
| Yoo 2011 |  | [31] | Serum | Korea | T2DM | Lower level of omentin-1 was observed in patients with diabetes mellitus than in the controls |
| Zhang 2014 | | [32] | Serum | China | T2DM | Lower level of omentin-1 was observed in patients with diabetes mellitus than in the controls |
| Abd El Dayem 2015 [33] | | | Serum | Egypt | T1DM | Lower level of omentin-1 was observed in patients with diabetes mellitus than in the controls |
| Nassif 2013 | | [34] | Serum | Egypt | T2DM | There was no significant difference in Omentin1 level between diabetic patients and control group. |
| Matloch 2018 | | [35] | Serum | Czech | T2DM | Lower level of omentin-1 was observed in patients with diabetes mellitus than in the controls |
| Gürsoy 2010 | | [36] | Plasma | Turkey | T2DM | Lower level of omentin-1 was observed in patients with diabetes mellitus than in the controls |
| Flehmig 2014 | | [37] | Serum | Germany | T2DM | Higher level of omentin-1 was observed in patients with diabetes mellitus than in the controls |
| Dogan 2016 | | [38] | Serum | Turkey | T2DM | Lower level of omentin-1 was observed in patients with diabetes mellitus than in the controls |
| Komosinska-vassev 2019 [39] | | | Plasma | Poland | T2DM | Higher level of omentin-1 was observed in patients with diabetes mellitus than in the controls |
| Rathwa 2019 | | [40] | Plasma | India | T2DM | Lower level of omentin-1 was observed in patients with diabetes mellitus than in the controls |
| Tuttolomondo 2019 [41] | | | Serum | Italy | T2DM | Lower level of omentin-1 was observed in patients with diabetes mellitus than in the controls |
| Souvannavong-vilivong 2019 [42] | | | Serum | Thailand | GDM | Lower level of omentin-1 was observed in patients with diabetes mellitus than in the controls |
| Aminilari 2017 [43] | | |  | Serum | Canada | T2DM |
| Arman 2017 | | [44] | Serum | Turkey | T2DM | Combined exercise was efficient in increasing serum omentin-1 among women with T2DM |
| Arslan 2017 | | [45] | Serum | Turkey | T2DM | In type 2 diabetes mellitus patients using insulin there was a significant decrease in Omentin-1 levels compared with the initial results |
| Biscetti 2019 | | [46] | Serum | Italy | T2DM | Diet plus metformin treatment decreased the omentin levels in type 2 diabetes patients |
| Esteghamati 2013 | | [47] | Serum | Iran | T2DM | Omentin-1 is reduced in type 2 diabetic patients with peripheral artery disease and that omentin-1 levels are related to disease severity. |
| Kaushik 2018 | | [48] | Plasma | India | Prediabetics | After three months, metformin decreased omentin concentrations in T2DM |
| Moreno-navarrete 2011 [49] | | Serum | Spain | Impaired glucose tolerance | Concentration of omentin-1 decreased in impaired glucose tolerance. | Concentration of plasma omentin-1 decreased and insulin resistance increased in obese prediabetics compared to obese normoglycaemics and healthy controls. |
| Sperling 2016[50] | | |  | Serum | Poland | Impaired glucose tolerance |
| Lesná 2015 | | [51] | Serum | Czech Republic | T1DM | Concentration of omentin-1 decreased in impaired glucose tolerance. |

1. Abd-Elbaky AE, Abo-Elmatty DM, Mesbah NM, Ibrahim SM. Omentin and apelin concentrations in relation to obesity, diabetes mellitus type two, and cardiovascular diseases in Egyptian population. International Journal of Diabetes in Developing Countries. 2015;36(1):1-7.

2. Abdelraouf Korany M, Sonbol A, Mohamed Elgouhary S. Omentin-1 and diabetic retinopathy in type 2 diabetic patients. Alexandria Journal of Medicine. 2018;54(4):323-6. doi: 10.1016/j.ajme.2018.04.003.

3. Abell SK, Shorakae S, Harrison CL, Hiam D, Moreno-Asso A, Stepto NK, et al. The association between dysregulated adipocytokines in early pregnancy and development of gestational diabetes. Diabetes Metab Res Rev. 2017;33(8). Epub 2017/08/15. doi: 10.1002/dmrr.2926. PubMed PMID: 28806491.

4. Ahmed HH, Shousha WG, El-mezayen HA, Emara IA, Hassan ME. New Biomarkers as Prognostic Factors for Cardiovascular Complications in Type 2 Diabetic Patients. Indian Journal of Clinical Biochemistry. 2018. doi: 10.1007/s12291-018-0784-4.

5. Akbarzadeh S, Nabipour I, Assadi M, Movahed A, Jafari SM, Motamed N, et al. The normoglycemic first-degree relatives of patients with type 2 diabetes mellitus have low circulating omentin-1 and adiponectin levels. Cytokine. 2012;58(2):295-9. Epub 2012/03/09. doi: 10.1016/j.cyto.2012.02.005. PubMed PMID: 22398372.

6. Akour A, Kasabri V, Boulatova N, Bustanji Y, Naffa R, Hyasat D, et al. Levels of metabolic markers in drug-naive prediabetic and type 2 diabetic patients. Acta Diabetol. 2017;54(2):163-70. Epub 2016/10/19. doi: 10.1007/s00592-016-0926-1. PubMed PMID: 27752839.

7. Cai RC, Wei L, Wu HY, Bao YQ, Jia WP, Xiang KS. Serum omentin level and related factors in obese and type 2 diabetic patients. National Medical Journal of China. 2008;88(16):1096-9.

8. Dayem SM, Battah AA, Shehaby AE. Cardiac Affection in Type 1 Diabetic Patients in Relation to Omentin. Open Access Maced J Med Sci. 2015;3(4):699-704. Epub 2016/06/09. doi: 10.3889/oamjms.2015.132. PubMed PMID: 27275311; PubMed Central PMCID: PMCPMC4877911.

9. El-Mesallamy HO, El-Derany MO, Hamdy NM. Serum omentin-1 and chemerin levels are interrelated in patients with Type 2 diabetes mellitus with or without ischaemic heart disease. Diabet Med. 2011;28(10):1194-200. Epub 2011/06/15. doi: 10.1111/j.1464-5491.2011.03353.x. PubMed PMID: 21668495.

10. Elsaid NH, Sadik NA, Ahmed NR, Fayez SE, Mohammed NAE. Serum omentin-1 levels in type 2 diabetic obese women in relation to glycemic control, insulin resistance and metabolic parameters. J Clin Transl Endocrinol. 2018;13:14-9. Epub 2018/07/20. doi: 10.1016/j.jcte.2018.05.003. PubMed PMID: 30023310; PubMed Central PMCID: PMCPMC6047309.

11. Franz M, Polterauer M, Springer S, Kuessel L, Haslinger P, Worda C, et al. Maternal and neonatal omentin-1 levels in gestational diabetes. Arch Gynecol Obstet. 2018;297(4):885-9. Epub 2018/01/18. doi: 10.1007/s00404-018-4652-5. PubMed PMID: 29335783; PubMed Central PMCID: PMCPMC5849644.

12. Greulich S, Chen WJ, Maxhera B, Rijzewijk LJ, van der Meer RW, Jonker JT, et al. Cardioprotective properties of omentin-1 in type 2 diabetes: evidence from clinical and in vitro studies. PLoS One. 2013;8(3):e59697. Epub 2013/04/05. doi: 10.1371/journal.pone.0059697. PubMed PMID: 23555749; PubMed Central PMCID: PMCPMC3612072.

13. Hayashi M, Morioka T, Hatamori M, Kakutani Y, Yamazaki Y, Kurajoh M, et al. Plasma omentin levels are associated with vascular endothelial function in patients with type 2 diabetes at elevated cardiovascular risk. Diabetes Res Clin Pract. 2019;148:160-8. Epub 2019/01/15. doi: 10.1016/j.diabres.2019.01.009. PubMed PMID: 30641171.

14. Herder C, Bongaerts BW, Ouwens DM, Rathmann W, Heier M, Carstensen-Kirberg M, et al. Low serum omentin levels in the elderly population with Type 2 diabetes and polyneuropathy. Diabet Med. 2015;32(11):1479-83. Epub 2015/06/23. doi: 10.1111/dme.12761. PubMed PMID: 26094489.

15. Kahwaji R, Kasabri V, Bulatova N, Akour A, Bustanji H, Khawaja N, et al. Evaluation of correlations of Plasma Levels of Oxytocin, Omentin-1 and Irisin in Diabetic and Non-Diabetic Metabolic Syndrome Patients: A Cross Sectional Study in Jordan. Jordan Medical Journal. 2017;51(3):97-108.

16. Kocijancic M, Vujicic B, Racki S, Cubranic Z, Zaputovic L, Dvornik S. Serum omentin-1 levels as a possible risk factor of mortality in patients with diabetes on haemodialysis. Diabetes Res Clin Pract. 2015;110(1):44-50. Epub 2015/08/22. doi: 10.1016/j.diabres.2015.06.008. PubMed PMID: 26293449.

17. Lewandowski K, Nadel I, Lewinski A, Bienkiewicz M, Tan B, Randeva HS, et al. Positive correlation between serum omentin and thrombospondin-1 in gestational diabetes despite lack of correlation with insulin resistance indices. Ginekol Pol. 2010;81(12):907-12. Epub 2011/03/12. PubMed PMID: 21391440.

18. Madsen SM, Thorup AC, Bjerre M, Jeppesen PB. Does 8 weeks of strenuous bicycle exercise improve diabetes-related inflammatory cytokines and free fatty acids in type 2 diabetes patients and individuals at high-risk of metabolic syndrome? Arch Physiol Biochem. 2015;121(4):129-38. Epub 2015/10/16. doi: 10.3109/13813455.2015.1082600. PubMed PMID: 26469542.

19. Mierzynski R, Dluski D, Nowakowski L, Poniedzialek-Czajkowska E, Leszczynska-Gorzelak B. Adiponectin and Omentin Levels as Predictive Biomarkers of Preterm Birth in Patients with Gestational Diabetes Mellitus. Biomed Res Int. 2018;2018:7154216. Epub 2018/10/16. doi: 10.1155/2018/7154216. PubMed PMID: 30320137; PubMed Central PMCID: PMCPMC6167585.

20. Motawi TMK, Mahdy SG, El-Sawalhi MM, Ali EN, El-Telbany RFA. Serum levels of chemerin, apelin, vaspin, and omentin-1 in obese type 2 diabetic Egyptian patients with coronary artery stenosis. Canadian journal of physiology and pharmacology. 2018;96(1):38-44. doi: 10.1139/cjpp-2017-0272.

21. Nurten E, Vogel M, Michael Kapellen T, Richter S, Garten A, Penke M, et al. Omentin-1 and NAMPT serum concentrations are higher and CK-18 levels are lower in children and adolescents with type 1 diabetes when compared to healthy age, sex and BMI matched controls. J Pediatr Endocrinol Metab. 2018;31(9):959-69. Epub 2018/09/05. doi: 10.1515/jpem-2018-0353. PubMed PMID: 30179852.

22. Pan HY, Guo L, Li Q. Changes of serum omentin-1 levels in normal subjects and in patients with impaired glucose regulation and with newly diagnosed and untreated type 2 diabetes. Diabetes Res Clin Pract. 2010;88(1):29-33. Epub 2010/02/05. doi: 10.1016/j.diabres.2010.01.013. PubMed PMID: 20129687.

23. Polkowska A, Szczepaniak I, Bossowski A. Assessment of Serum Concentrations of Ghrelin, Obestatin, Omentin-1, and Apelin in Children with Type 1 Diabetes. Biomed Res Int. 2016;2016:8379294. Epub 2016/02/24. doi: 10.1155/2016/8379294. PubMed PMID: 26904686; PubMed Central PMCID: PMCPMC4745415.

24. Tan BK, Pua S, Syed F, Lewandowski KC, O'Hare JP, Randeva HS. Decreased plasma omentin-1 levels in Type 1 diabetes mellitus. Diabet Med. 2008;25(10):1254-5. Epub 2008/12/03. doi: 10.1111/j.1464-5491.2008.02568.x. PubMed PMID: 19046210.

25. Tekce H, Tekce BK, Aktas G, Alcelik A, Sengul E. Serum omentin-1 levels in diabetic and nondiabetic patients with chronic kidney disease. Exp Clin Endocrinol Diabetes. 2014;122(8):451-6. Epub 2014/06/12. doi: 10.1055/s-0034-1375674. PubMed PMID: 24918534.

26. Tsiotra PC, Halvatsiotis P, Patsouras K, Maratou E, Salamalekis G, Raptis SA, et al. Circulating adipokines and mRNA expression in adipose tissue and the placenta in women with gestational diabetes mellitus. Peptides. 2018;101:157-66. doi: 10.1016/j.peptides.2018.01.005.

27. Urbanová M, Dostálová I, Trachta P, Drápalová J, Kaválková P, Haluzíková D, et al. Serum concentrations and subcutaneous adipose tissue mRNA expression of omentin in morbid obesity and type 2 diabetes mellitus: the effect of very-low-calorie diet, physical activity and laparoscopic sleeve gastrectomy. Physiological research / Academia Scientiarum Bohemoslovaca. 2014;63(2):207-18.

28. Wan W, Li Q, Zhang F, Zheng G, Lv Y, Wan G, et al. Serum and Vitreous Concentrations of Omentin-1 in Diabetic Retinopathy. Dis Markers. 2015;2015:754312. Epub 2015/10/01. doi: 10.1155/2015/754312. PubMed PMID: 26420914; PubMed Central PMCID: PMCPMC4573431.

29. Yan P, Li L, Yang M, Liu D, Liu H, Boden G, et al. Effects of the long-acting human glucagon-like peptide-1 analog liraglutide on plasma omentin-1 levels in patients with type 2 diabetes mellitus. Diabetes Res Clin Pract. 2011;92(3):368-74. Epub 2011/04/05. doi: 10.1016/j.diabres.2011.02.030. PubMed PMID: 21458097.

30. Yan P, Liu D, Long M, Ren Y, Pang J, Li R. Changes of serum omentin levels and relationship between omentin and adiponectin concentrations in type 2 diabetes mellitus. Exp Clin Endocrinol Diabetes. 2011;119(4):257-63. Epub 2011/03/05. doi: 10.1055/s-0030-1269912. PubMed PMID: 21374544.

31. Yoo HJ, Hwang SY, Hong HC, Choi HY, Yang SJ, Seo JA, et al. Association of circulating omentin-1 level with arterial stiffness and carotid plaque in type 2 diabetes. Cardiovasc Diabetol. 2011;10:103. Epub 2011/11/24. doi: 10.1186/1475-2840-10-103. PubMed PMID: 22108456; PubMed Central PMCID: PMCPMC3235986.

32. Zhang Q, Zhu L, Zheng M, Fan C, Li Y, Zhang D, et al. Changes of serum omentin-1 levels in normal subjects, type 2 diabetes and type 2 diabetes with overweight and obesity in Chinese adults. Ann Endocrinol (Paris). 2014;75(3):171-5. Epub 2014/07/07. doi: 10.1016/j.ando.2014.04.013. PubMed PMID: 24997770.

33. Eldayem SMA, Battah AA, Elshehaby A. Cardiac affection in type 1 diabetic patients in relation to omentin. Open Access Macedonian Journal of Medical Sciences. 2015;3(4):699-704.

34. Nassif WMH, Amin AI, Hassan ZA. Changes of serum omentin-1 levels and relationship between omentin-1 and insulin resistance in chronic hepatitis C patients. Excli Journal. 2013;12:924-32.

35. Matloch Z, Kratochvilova H, Cinkajzlova A, Lips M, Kopecky P, Porizka M, et al. Changes in omentin levels and its mRNA expression in epicardial adipose tissue in patients undergoing elective cardiac surgery: the influence of type 2 diabetes and coronary heart disease. Physiol Res. 2018;67(6):881-90. Epub 2018/09/12. doi: 10.33549/physiolres.933909. PubMed PMID: 30204471.

36. Gürsoy G, Kırnap NG, Ebah O, Acar Y, Demirba B, Akçayöz S, et al. The relationship between plasma omentin-1 levels and insulin resistance in newly diagnosed type 2 diabetıc women. Clinical Reviews & Opinions. 2010;(4):49-54.

37. Flehmig G, Scholz M, Klã¶Ting N, Fasshauer M, Tã¶Njes A, Stumvoll M, et al. Identification of adipokine clusters related to parameters of fat mass, insulin sensitivity and inflammation. Plos One. 2014;9(6):e99785.

38. Bozkurt Doğan Ş, Öngöz Dede F, Ballı U, Sertoğlu E. Levels of vaspin and omentin-1 in gingival crevicular fluid as potential markers of inflammation in patients with chronic periodontitis and type 2 diabetes mellitus. Journal of Oral Science. 2016;58(3):379-89. doi: 10.2334/josnusd.15-0731.

39. Komosinska-Vassev K, Olczyk P, Kuznik-Trocha K, Jura-Poltorak A, Derkacz A, Purchalka M, et al. Circulating C1q/TNF-Related Protein 3, Omentin-1 and NGAL in Obese Patients with Type 2 Diabetes During Insulin Therapy. Journal of clinical medicine. 2019;8(6). Epub 2019/06/15. doi: 10.3390/jcm8060805. PubMed PMID: 31195747; PubMed Central PMCID: PMCPMC6617185.

40. Rathwa N, Patel R, Palit SP, Jadeja SD, Narwaria M, Ramachandran AV, et al. Circulatory Omentin-1 levels but not genetic variants influence the pathophysiology of Type 2 diabetes. Cytokine. 2019;119:144-51. doi: 10.1016/j.cyto.2019.03.011. PubMed PMID: WOS:000471356000018.

41. Tuttolomondo A, Di Raimondo D, Casuccio A, Guercio G, Del Cuore A, Puleo MG, et al. Endothelial function, adipokine serum levels and white matter hyperintesities in subjects with diabetic foot syndrome. The Journal of clinical endocrinology and metabolism. 2019. doi: 10.1210/jc.2018-02507.

42. Souvannavong-Vilivong X, Sitticharoon C, Klinjampa R, Keadkraichaiwat I, Sripong C, Chatree S, et al. Placental expressions and serum levels of adiponectin, visfatin, and omentin in GDM. Acta Diabetologica. 2019. doi: 10.1007/s00592-019-01355-0.

43. AminiLari Z, Fararouei M, Amanat S, Sinaei E, Dianatinasab S, AminiLari M, et al. The Effect of 12 Weeks Aerobic, Resistance, and Combined Exercises on Omentin-1 Levels and Insulin Resistance among Type 2 Diabetic Middle-Aged Women. Diabetes Metab J. 2017;41(3):205-12. Epub 2017/05/26. doi: 10.4093/dmj.2017.41.3.205. PubMed PMID: 28537059; PubMed Central PMCID: PMCPMC5489501.

44. Arman Y, Kirna K, Ugurlukisi B, Kutlu O, Dikker O, Cil EO, et al. The Effects of Blood Glucose Regulation in Omentin-1 Levels among Diabetic Patients. Exp Clin Endocrinol Diabetes. 2017;125(4):262-6. Epub 2017/01/11. doi: 10.1055/s-0042-118862. PubMed PMID: 28073124.

45. Arslan I, Ulas T, Karakas EY, Demir M, Eren MA, Torun A, et al. Comparative effectiveness of diet alone and diet plus metformin treatment on omentin levels in type 2 diabetes patients with nonalcoholic fatty liver disease: a prospective randomized trial. Periodicum biologorum. 2017;119(1):9‐15. doi: 10.18054/pb.v119i1.4180. PubMed PMID: CN-01374760.

46. Biscetti F, Nardella E, Bonadia N, Angelini F, Pitocco D, Santoliquido A, et al. Association between plasma omentin-1 levels in type 2 diabetic patients and peripheral artery disease. Cardiovascular Diabetology. 2019;18(1). doi: 10.1186/s12933-019-0880-7.

47. Esteghamati A, Noshad S, Rabizadeh S, Ghavami M, Zandieh A, Nakhjavani M. Comparative effects of metformin and pioglitazone on omentin and leptin concentrations in patients with newly diagnosed diabetes: a randomized clinical trial. Regul Pept. 2013;182:1-6. Epub 2013/01/19. doi: 10.1016/j.regpep.2012.12.005. PubMed PMID: 23328000.

48. Kaushik N, Kaushik R, Dixit P, Tyagi M, Gambhir J, Madhu SV, et al. Plasma Omentin-1 Level and its Relationship with Insulin Resistance in Obese Prediabetics. JOURNAL OF CLINICAL AND DIAGNOSTIC RESEARCH. 2018;12. doi: 10.7860/JCDR/2018/31845.11432.

49. Moreno-Navarrete JM, Ortega F, Castro A, Sabater M, Ricart W, Fernandez-Real JM. Circulating omentin as a novel biomarker of endothelial dysfunction. Obesity (Silver Spring, Md). 2011;19(8):1552-9. Epub 2011/02/05. doi: 10.1038/oby.2010.351. PubMed PMID: 21293447.

50. Sperling M, Grzelak T, Pelczynska M, Jasinska P, Bogdanski P, Pupek-Musialik D, et al. Concentrations of omentin and vaspin versus insulin resistance in obese individuals. Biomed Pharmacother. 2016;83:542-7. Epub 2016/10/25. doi: 10.1016/j.biopha.2016.07.012. PubMed PMID: 27449535.

51. Lesna J, Ticha A, Hyspler R, Musil F, Blaha V, Sobotka L, et al. Omentin-1 plasma levels and cholesterol metabolism in obese patients with diabetes mellitus type 1: impact of weight reduction. Nutr Diabetes. 2015;5:e183. Epub 2015/11/03. doi: 10.1038/nutd.2015.33. PubMed PMID: 26524638; PubMed Central PMCID: PMCPMC4631935.
