# Supplementary material for: Omentin-1 in diabetes mellitus: A systematic review and meta-analysis
Source: PLoS One. 2019 Dec 10;14(12):e0226292. doi: 10.1371/journal.pone.0226292 (PMC6903756; doi:10.1371/journal.pone.0226292)
Supplement: S4 Table — (DOC) [file pone.0226292.s004.doc]

**NEWCASTLE - OTTAWA QUALITY ASSESSMENT SCALE**

**🟑**This symbol represents the option and gets one point.

**Selection**

1) Is the case definition adequate?

a) yes, with independent validation **🟑**

b) yes, eg record linkage or based on self reports

c) no description

2) Representativeness of the cases

a) consecutive or obviously representative series of cases **🟑**

b) potential for selection biases or not stated

3) Selection of Controls

a) community controls **🟑**

b) hospital controls

c) no description

4) Definition of Controls

a) no history of disease (endpoint) **🟑**

b) no description of source

**Comparability**

5.6) Comparability of cases and controls on the basis of the design or analysis

a) study controls the most important factor. **🟑**

b) study controls for any additional factor **🟑**

**Outcome**

7) Ascertainment of exposure

a) secure record (eg surgical records) **🟑**

b) structured interview where blind to case/control status **🟑**

c) interview not blinded to case/control status

d) written self report or medical record only

e) no description

8) Same method of ascertainment for cases and controls

a) yes **🟑**

b) no

9) Non-Response rate

a) same rate for both groups **🟑**

b) non respondents described

c) rate different and no designation

**The NOS Score Scale**

| **Study** | **Total score** | **Selection** | | | | **Comparability** | | **Outcome** | | |
| --- | --- | --- | --- | --- | --- | --- | --- | --- | --- | --- |
| **Item** |  | **1** | **2** | **3** | **4** | **5** | **6** | **7** | **8** | **9** |
| Abd-elbaky 2015 | 7 | 1 | 1 | 1 | 1 | 1 | 0 | 1 | 1 | 0 |
| Abdelraoufkorany 2018 | 6 | 1 | 0 | 1 | 1 | 1 | 0 | 1 | 1 | 0 |
| Abell 2017 | 7 | 1 | 1 | 1 | 1 | 1 | 0 | 1 | 1 | 0 |
| Ahmed 2018 | 8 | 1 | 1 | 1 | 1 | 1 | 1 | 1 | 1 | 0 |
| Akbarzadeh 2012 | 7 | 1 | 1 | 1 | 1 | 1 | 0 | 1 | 1 | 0 |
| Akour 2016 | 8 | 1 | 1 | 1 | 1 | 1 | 1 | 1 | 1 | 0 |
| Cai 2008 | 8 | 1 | 1 | 1 | 1 | 1 | 1 | 1 | 1 | 0 |
| Dayem 2015 | 6 | 1 | 0 | 1 | 1 | 1 | 0 | 1 | 1 | 0 |
| El-mesallamy 2011 | 7 | 1 | 0 | 1 | 1 | 1 | 1 | 1 | 1 | 0 |
| Elsaid 2018 | 8 | 1 | 1 | 1 | 1 | 1 | 1 | 1 | 1 | 0 |
| Franz 2018 | 7 | 1 | 1 | 1 | 1 | 1 | 0 | 1 | 1 | 0 |
| Greulich 2013 | 7 | 1 | 1 | 1 | 1 | 1 | 0 | 1 | 1 | 0 |
| Hayashi 2018 | 5 | 1 | 0 | 0 | 1 | 1 | 0 | 1 | 1 | 0 |
| Herder 2017 | 6 | 1 | 0 | 1 | 1 | 1 | 0 | 1 | 1 | 0 |
| Kahwaji 2017 | 7 | 1 | 1 | 1 | 1 | 1 | 0 | 1 | 1 | 0 |
| Kocijancic 2015 | 6 | 1 | 0 | 1 | 1 | 1 | 0 | 1 | 1 | 0 |
| Lewandowski 2010 | 6 | 1 | 0 | 1 | 1 | 1 | 0 | 1 | 1 | 0 |
| Madsen 2015 | 7 | 1 | 1 | 1 | 1 | 1 | 0 | 1 | 1 | 0 |
| Mierzyński 2018 | 7 | 1 | 1 | 0 | 1 | 1 | 1 | 1 | 1 | 0 |
| Motawi 2017 | 6 | 1 | 0 | 1 | 1 | 1 | 0 | 1 | 1 | 0 |
| Nurten 2018 | 7 | 1 | 1 | 1 | 1 | 1 | 0 | 1 | 1 | 0 |
| Pan 2010 | 7 | 1 | 1 | 0 | 1 | 1 | 1 | 1 | 1 | 0 |
| Polkowska 2016 | 6 | 1 | 1 | 0 | 1 | 1 | 0 | 1 | 1 | 0 |
| Tan 2008 | 6 | 1 | 0 | 1 | 1 | 1 | 0 | 1 | 1 | 0 |
| Tekce 2014 | 7 | 1 | 1 | 1 | 1 | 1 | 0 | 1 | 1 | 0 |
| Tsiotra 2018 | 7 | 1 | 1 | 1 | 1 | 1 | 0 | 1 | 1 | 0 |
| Urbanova 2014 | 6 | 1 | 0 | 1 | 1 | 1 | 0 | 1 | 1 | 0 |
| Wan 2015 | 7 | 1 | 1 | 1 | 1 | 1 | 0 | 1 | 1 | 0 |
| Yan 2011A | 7 | 1 | 1 | 1 | 1 | 1 | 0 | 1 | 1 | 0 |
| Yan 2011B | 7 | 1 | 1 | 1 | 1 | 1 | 0 | 1 | 1 | 0 |
| Yoo 2011 | 7 | 1 | 0 | 1 | 1 | 1 | 1 | 1 | 1 | 0 |
| Zhang 2014 | 8 | 1 | 1 | 1 | 1 | 1 | 1 | 1 | 1 | 0 |
| Abd El Dayem 2015 | 7 | 1 | 1 | 1 | 1 | 1 | 0 | 1 | 1 | 0 |
| Nassif 2013 | 5 | 1 | 0 | 0 | 1 | 1 | 0 | 1 | 1 | 0 |
| Matloch 2018 | 5 | 1 | 0 | 0 | 1 | 1 | 0 | 1 | 1 | 0 |
| Gürsoy 2010 | 6 | 1 | 0 | 1 | 1 | 1 | 0 | 1 | 1 | 0 |
| Flehmig 2014 | 7 | 1 | 1 | 1 | 1 | 1 | 0 | 1 | 1 | 0 |
| Dogan 2016 | 5 | 1 | 0 | 0 | 1 | 1 | 0 | 1 | 1 | 0 |
| Komosinska-vassev 2019 | 7 | 1 | 1 | 1 | 1 | 1 | 0 | 1 | 1 | 0 |
| Rathwa 2019 | 7 | 1 | 1 | 1 | 1 | 1 | 0 | 1 | 1 | 0 |
| Tuttolomondo 2019 | 6 | 1 | 1 | 0 | 1 | 1 | 0 | 1 | 1 | 0 |
| Souvannavong-vilivong 2019 | 6 | 1 | 0 | 1 | 1 | 1 | 0 | 1 | 1 | 0 |
